# An Overview of Insulin Pumps and Glucose Sensors for the Generalist

**DOI:** 10.3390/jcm5010005

**Published:** 2016-01-04

**Authors:** Brooke H. McAdams, Ali A. Rizvi

**Affiliations:** 1 Fellow in Endocrinology, University of South Carolina School of Medicine, Columbia, SC 29203, USA; brookeshollins@yahoo.com; 2 Medicine and Director, Endocrinology Division, University of South Carolina School of Medicine, Two Medical Park, Suite 502, Columbia, SC 29203, USA

**Keywords:** diabetes, insulin pump, pancreas, glucose sensor, monitoring

## Abstract

Continuous subcutaneous insulin, or the insulin pump, has gained popularity and sophistication as a near-physiologic programmable method of insulin delivery that is flexible and lifestyle-friendly. The introduction of continuous monitoring with glucose sensors provides unprecedented access to, and prediction of, a patient’s blood glucose levels. Efforts are underway to integrate the two technologies, from “sensor-augmented” and “sensor-driven” pumps to a fully-automated and independent sensing-and-delivery system. Implantable pumps and an early-phase “bionic pancreas” are also in active development. Fine-tuned “pancreas replacement” promises to be one of the many avenues that offers hope for individuals suffering from diabetes. Although endocrinologists and diabetes specialists will continue to maintain expertise in this field, it behooves the primary care physician to have a working knowledge of insulin pumps and sensors to ensure optimal clinical care and decision-making for their patients.

## 1. Introduction

Diabetes is rapidly becoming a major health epidemic in most regions of the world [1]. All patients with type 1 diabetes and a significant number with type 2 diabetes require the use of insulin for controlling blood glucose. In the last 20 years, technological innovation and bioengineering has transformed the diabetes therapeutic landscape. There are several varieties of insulin and many different injection regimens that can be used. However, in spite of the availability of insulin vials and pens, the acceptability for patients and the glucose readings that are obtained with the use of single or multiple-dose injection regimens is not to the desired level. Insulin delivery with pumps, also known as *continuous subcutaneous insulin infusion (CSII)*, was introduced almost a half century ago. It utilizes short- or rapid-acting insulin types only, thus minimizing variability of administration and reducing the chances of glucose fluctuations. Pump technology has progressed to the level of precisely mimicking physiological demands. Programmable insulin administration in basal and bolus fashion is integrated and augmented with glucose biosensors to provide real-time, data-driven glycemic control and early detection of hypoglycemia. The prospect of a functional, closed-loop “artificial pancreas” with implantable or bionic capabilities is now within the realm of technological possibility in the near future.

## 2. How Do Insulin Pumps Work?

Insulin pumps deliver insulin by continuous infusion through a single subcutaneous site which is replaced, on average, every three days. Only rapid-acting insulin is used, and the analogue insulins have gained popularity over regular insulin for this purpose [2]. A pump delivers programmable *basal insulin* around the clock which is tailored to the patient’s 24-h glucose profile. The insulin requirements may be affected by the individual’s physiology, the type and duration of daily activity, work schedule, exercise, illness, concomitant medications, *etc*. Most patients utilize multiple basal rates over a 24-h period, but some may use a single rate. Almost all pumps have the capability of programming basal rates that are modifiable every hour and also have a temporary basal rate feature for special situations. Patients can also deliver *bolus insulin* which infuses over a few minutes to a few hours. Insulin boluses cover meals and correct for high blood glucose levels. For the pump to accurately calculate bolus insulin amounts, the carbohydrate content of food and the blood glucose level are required. Insulin delivery via the pump can be suspended by the patient if necessary.

## 3. Evolution of Insulin Pump Technology

**Figure 1:**
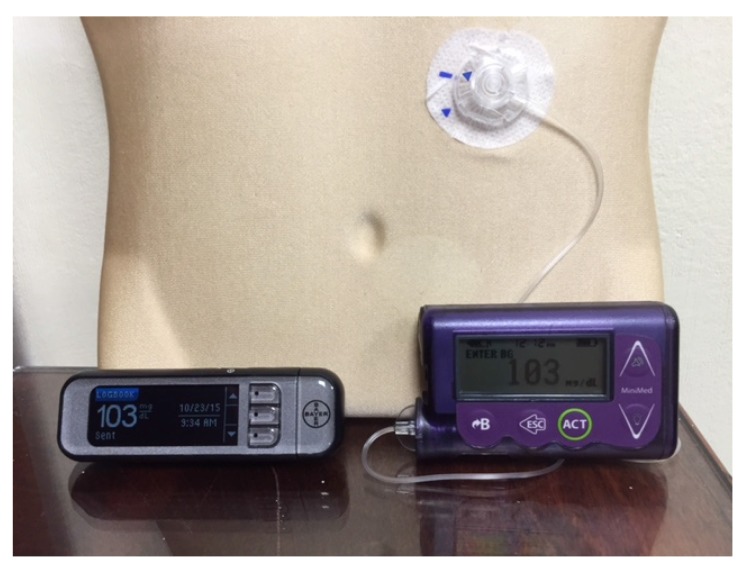
A Medtronic Minimed Insulin Pump and a blood glucose meter that communicates blood glucose readings wirelessly with it.

**Figure 2:**
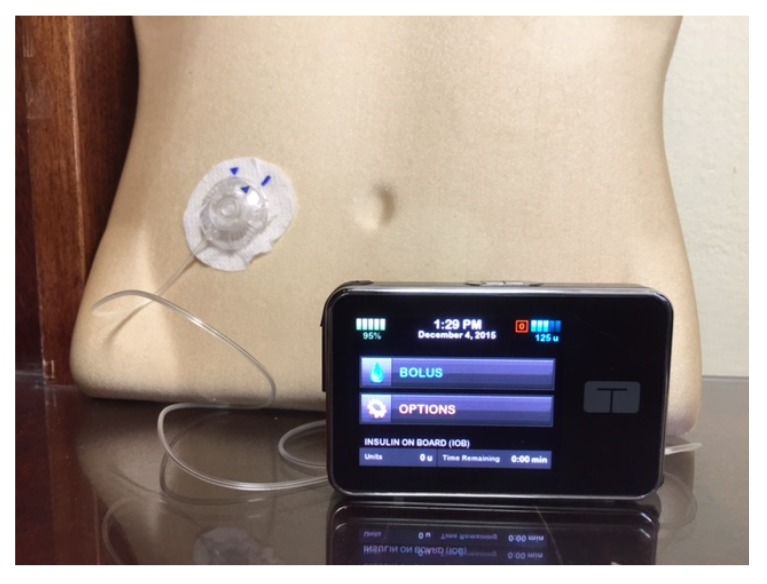
The T-slim Insulin Pump is popular with young patients due to its new touch-screen design.

**Figure 3:**
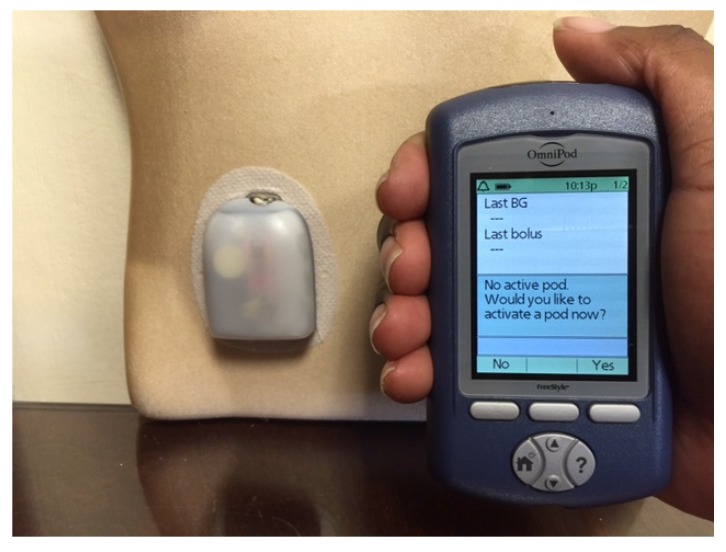
The OmniPod Tubeless Insulin Pump with a pod (**right**) and a handheld device that functions as a blood glucose meter and communicates wirelessly with the pod to deliver insulin based on the patient’s personal settings.

**Figure 4:**
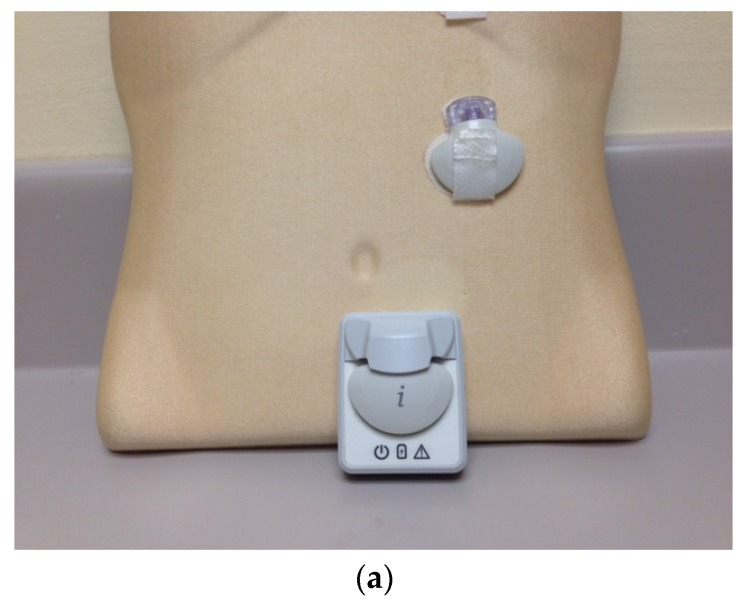

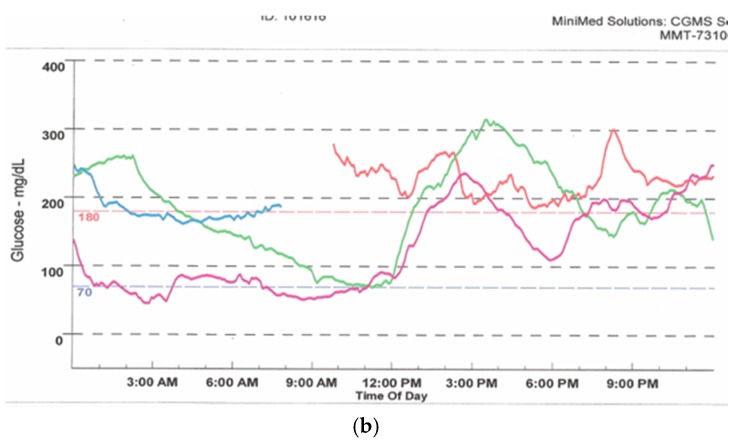
The Medtronic iPro2 Professional Continuous Glucose Monitor (**a**) with its charger, and a Downloaded Tracing showing daily color-coded readings (**b**).

The first insulin pumps were bulky machines that were employed only for research purposes. Pumps were commercialized for use in the general diabetic patient population in the 1970s. They required patients to calculate the amount of bolus insulin for food coverage and for high glucose readings [3]. Technological capabilities have advanced dramatically since that time, and pump use has increased manifold. Statistics show that an estimated 350,000 people in the United States (US) use insulin pumps today, and about 30,000 of those are believed to have Type 2 diabetes [4]. Currently available pumps deliver basal insulin in increments of as little as 0.01 units per hour, and use automatic bolus insulin calculators. Although the pump and its supplies (insulin cartridges, tubing, and infusion sets) remain expensive, insurance coverage has improved considerably over the years. With phenomenal advances in self-monitoring of blood glucose, meters are able to communicate the readings directly and wirelessly to the pump via infrared technology, thus eliminating the extra step of manual entry of the glucose value into the pump by the patient. The Medtronic Minimed and the Animas Vibe brands are examples of some of the more commonly prescribed insulin pumps (Figure 1 and Figure 2). Touch screen technology and user-friendliness have been incorporated in Tandem diabetes Care’s T-Slim insulin pump (Figure 3). Tubeless and fully disposable pumps provide a desirable option for many patients (OmniPod Insulin Pump, Figure 4). On average, an insulin pump costs about US $6000 and supplies between US $3000 and $6000 annually. Patients who switch from multiple-dose insulin injections (MDII) to pumps in a managed care setting realize a reduction in insulin expenditures of around US $657 per year [5].

## 4. Potential Benefits of Pump Therapy

The expected benefits of insulin pump therapy are summarized in Table 1. Insulin pump use provides a near-physiologic basal-bolus insulin delivery method that, in theory, mimics normal pancreatic function better than injections [6]. Precise insulin dosages can be programmed and administered, giving the patient increased flexibility in daily living with regard to mealtimes, travel, work schedule, *etc.* [7]. Observational studies, meta-analyses, and randomized clinical trials have demonstrated improvements in long-term glycemic control when compared with daily multiple-dose insulin injections [8,9,10]. Evidence from clinical studies suggests that pump therapy is associated with a decreased risk of severe hypoglycemia and the need for emergent medical care [11]. The latter translates into reduction in the cost of care and utilization of health care resources [12]. Quality of life measures have shown improvement with pump therapy compared with MDII [13].

**Table 1:** Potential Advantages of Continuous Subcutaneous Insulin Infusion (Insulin Pump Therapy) when compared to Multiple-Dose Insulin Injections.

Programmable insulin delivery allows closer match with physiologic needs
Uses only short- or rapid-acting insulin, minimizing peaks and absorption-related variability
Uses one injection site for up to 72 h, thus reducing variations in absorption and treatment-related burden from multiple injections
Reduction in glycemic variability and improved glycemic control
Decreased risk of severe hypoglycemia and need for emergent medical attention
Reduction in need for hospitalization and cost of care
Improved quality of life and treatment satisfaction

Issues unique to pumps are related to mechanical malfunction and insertion site issues such as inflammation or, rarely, skin infections [14]. Interruption of insulin supply due to kinking or blockage of the infusion set can lead to the rapid development of extreme hyperglycemia, especially in type 1 diabetes [15]. These factors may sometimes necessitate discontinuation of pump use and temporary reversion to insulin injections. Safeguards such as alarms that warn of delivery problems or low amounts of insulin in the reservoir are now standard features in insulin pumps.

## 5. Selection of Candidates for Pump Use

The common situations in which insulin pump therapy may be considered are summarized in Table 2. The ideal candidate for initiation of insulin pump therapy is a motivated patient who is knowledgeable in the important aspects of diabetes self-care and desires better glycemic control [16] (Table 3). The patient should be familiar with carbohydrate counting and CSII technology, and harbor realistic expectations about the amount of effort required and the benefits to be accrued from switching to an insulin pump. A pump is neither a cure for diabetes nor does it function autonomously without intervention and input. Patients should be clear about the fact that a pump is a highly specialized gadget; it nevertheless requires programming and constant interaction from the wearer. Adherence to blood glucose self-monitoring and the ability and willingness to regularly communicate with the professional pump team is absolutely critical in predicting long-term success with the pump [17]. The benefits afforded by integrated pump-sensor technology should also be weighed against its complexity and cost [18].

**Table 2:** Indications for Insulin Pump Treatment.

Suboptimal glycemic control in spite of efforts multiple daily insulin injections
Frequent or unpredictable hypoglycemia and hypoglycemia unawareness
“Dawn” phenomenon with persistent early-morning hyperglycemia
An active lifestyle (exercise, strenuous physical activity, athletic pursuits)
Children and young adults who typically desire fewer restrictions and more flexibility
Growth spurt of adolescence
Preconception planning and pregnancy
Presence of gastroparesis
Hectic lifestyle and frequent travel
Shift work and erratic daily schedules
Need for flexibility in amount and timing of meals
Type 2 diabetes with increased insulin requirements

## 6. Continuous Glucose Monitoring (CGM)

**Figure 5:**
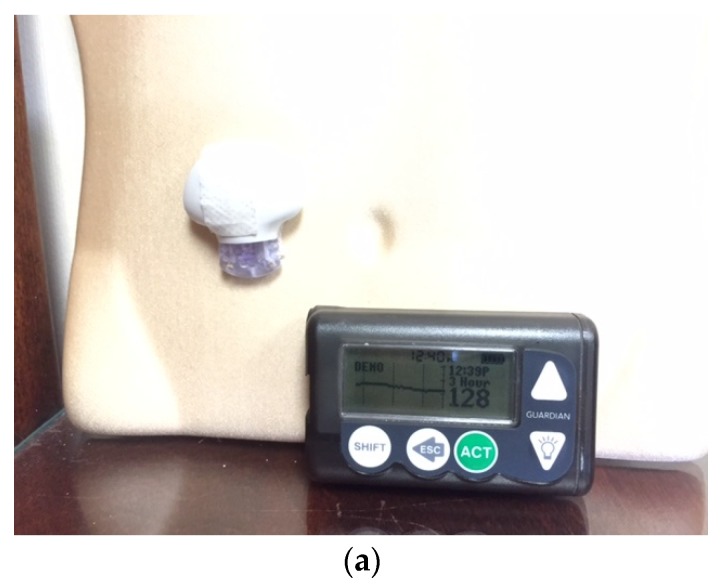

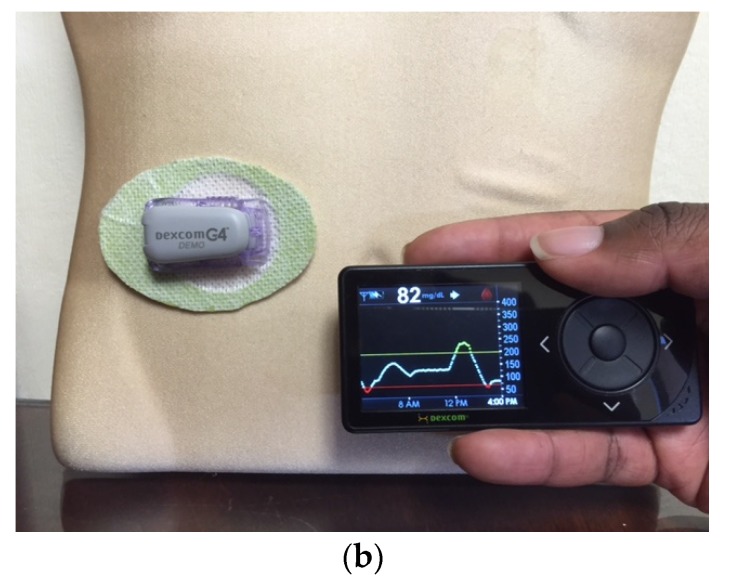
Personal Continuous Glucose Monitors: (**a**) the Medtronic *Guardian*; and (**b**) the *Dexcom-7*.

A landmark change in diabetes care came with the development of continuous glucose monitoring (CGM) via an indwelling subcutaneous sensor that could check interstitial fluid glucose readings every 3–5 min. CGM provides more frequent information on blood glucose less invasively than with the use of a traditional meter [19]. It also gives hourly, daily, and weekly glucose trends and patterns, and knowledge of glucose values in real time. Two types of continuous glucose monitoring systems are available. The *Professional CGM* works in a similar fashion to a cardiac Holter monitor. It can be used by physicians’ offices to record the patient’s ambulatory glucose values for up to six days, followed by downloading and retrospective review and analysis [20,21] (Figure 4). The *Personal CGM* is a patient-owned device that allows a small sensor placed subcutaneously to send glucose readings wirelessly and viewed on a separate receiver device (Figure 5) [22]. An individual sensor can stay in place for up to seven days at a time.

## 7. The Personal CGM Integrated with the Pump

**Figure 6:**
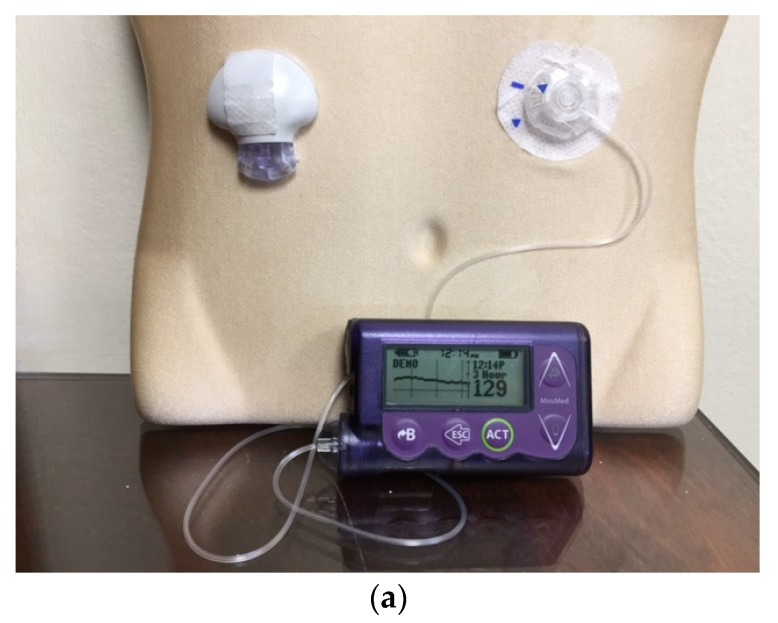

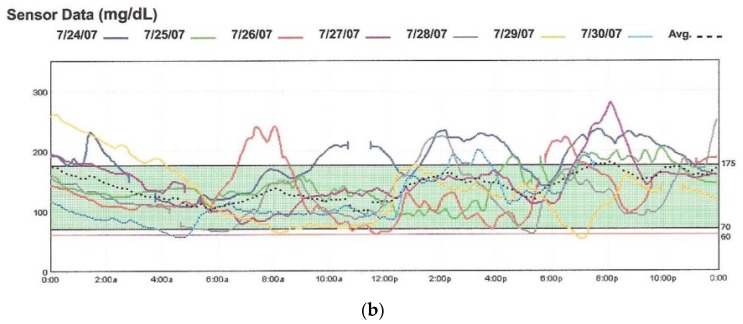
The *EnLite* Medtronic Real Time Continuous Glucose Sensor and Transmitter on the right in (**a**), integrated with the 530G Insulin Pump (Sensor-Augmented Pump Therapy). Downloaded 7-day tracing from the CareLink software are shown in (**b**).

**Figure 7:**
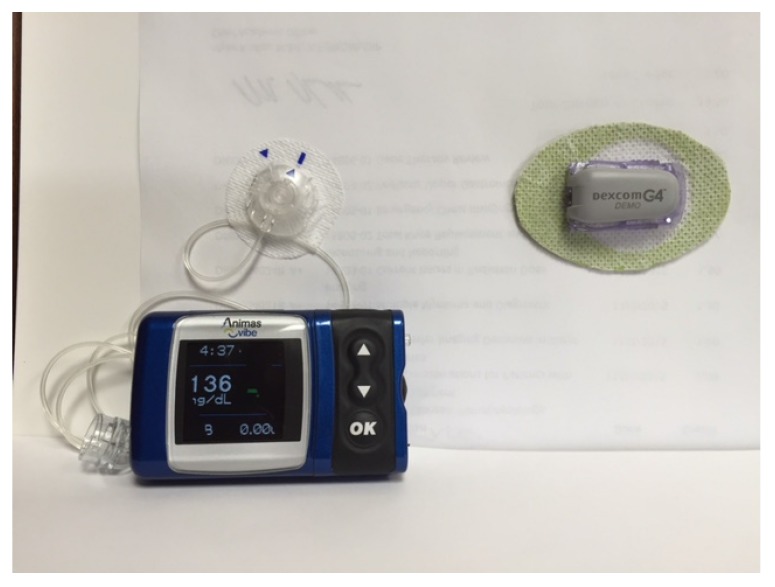
The Dexcom G4 Continuous Glucose Monitoring System (**left**) displays readings and graph on the screen of the Animas Vibe Insulin Pump.

As noted above, the Personal CGM is a patient-owned device that checks and displays glucose levels on a screen in real time and on a near-continuous basis. Although they can be used as independent gadgets, the *EnLite* Medtronic Continuous Glucose Sensor was developed specifically to integrate with an insulin pump (Medtronic 530G), thus displaying glucose readings on the screen of the pump without the need for a separate receiving device (Figure 6). The resultant integrated assembly gave rise to the popularity of sensor-augmented pump therapy which was shown to be superior to traditional daily multi-dose insulin injection use in several high-profile clinical trials [23,24]. It is equipped with alarms for high and low readings, as well as “Up” and “Down” trend arrows for rapidly changing values. More recently, the Animas *Vibe* pump has been configured to display glucose readings on its screen that it receives from the *Dexcom G4* CGM system (Figure 7).

**Figure 8:**
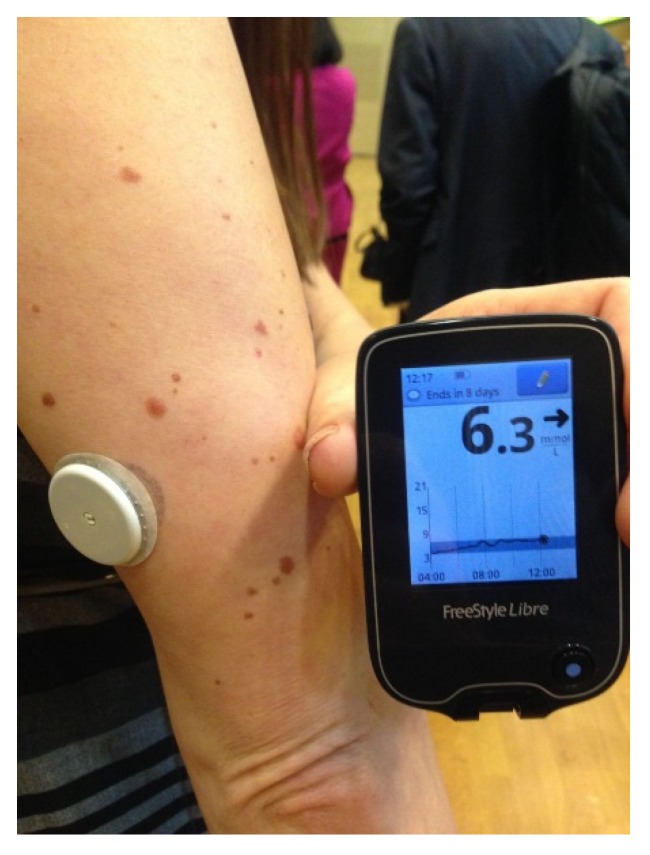
The Abbott FreeStyle Libre Flash Glucose Monitoring System (available online: http://diatribe.org/issues/69/new-now-next/1, accessed on 3 December 2015).

*The FreeStyle Libre Flash Glucose Monitoring System* introduced the concept of intermittent access to continuous glucose monitoring [25]. Marketed in Europe by Abbott Technologies in 2014, it consists of a tiny 0.5 cm glucose sensor inserted under the skin and connected to a water resistant, plastic coin-size skin patch. It is worn for 14 days and does not require fingerstick calibrations. A touchscreen reader device held near the sensor patch displays the real-time glucose value, a glucose trend arrow, and a trend graph showing the last eight hours of downloadable data (Figure 8). 

## 8. Sensor-Driven Insulin Delivery and “Auto-Shut-Off”

**Figure 9:**
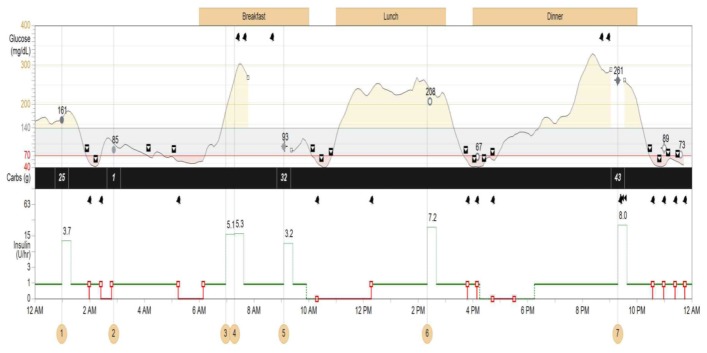
Download from a 530G Medtronic insulin pump with continuous glucose sensor (Enlite) and “Threshold Suspend” feature. The upper tracing shows multiple episodes of hypoglycemia with corresponding automatic suspension of insulin delivery (lower line) secondary to sensor-detected hypoglycemia.

The most recent advancement in insulin pump technology has been the first, albeit tiny, step towards the development of a genuine sensor-*driven* insulin pump. The Medtronic Veo and 530G pump brands represent the latest versions where an actual or predicted hypoglycemic value detected by a sensor that integrally communicates with the pump would cause the insulin delivery to be stopped—a form of threshold-based insulin-pump interruption—the so-called “Auto Shut-Off” feature (Figure 9) [26]. This mechanism does not require the patient’s intervention in order to interrupt the insulin delivery. It has been promoted as being a safety feature against hypoglycemia, especially during sleep or in patients who have hypoglycemia unawareness, as less time is spent in the hypoglycemic glucose range. More importantly, it demonstrates how a sensor-pump unit can be advanced from merely being data-providing gadgetry with alarms and cues, to actual modification and adjustment of insulin administration without patient input—an example of a partial *closed-loop system* or a “true artificial pancreas” [27]. Although an exciting development, the concept of a fully automated *sensing and delivering pump* is still evolving and can only be considered to be currently in its very early stages. Professional organizations such as the Endocrine Society have published guidelines on the current status and use of CGM in clinical practice [28].

## 9. Implantable Pumps

**Figure 10:**
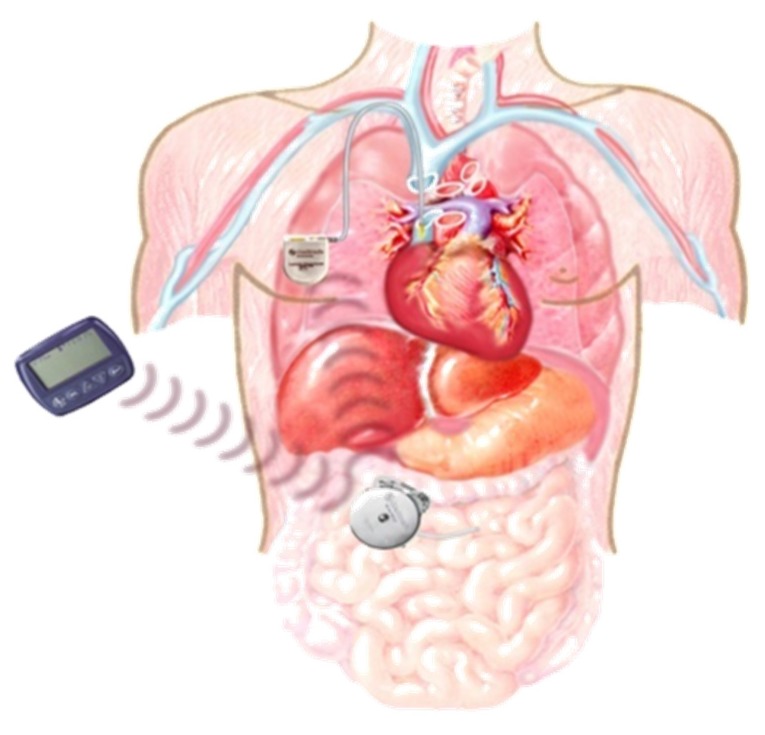
Diagrammatic representation of an implantable insulin pump (available online: https://thedishondiabetes.wordpress.com, accessed on 3 December 2015).

Efforts are also under way to implant longer-lasting pumps internally, such as in the intra-peritoneal cavity. An implantable insulin pump, possibly with a sensor and a digital handheld control device for receiving glucose readings and knowledge or manipulation of insulin delivery, would be discreet with ostensibly good patient acceptability (Figure 10). A subcutaneous side-port could be used for refilling concentrated insulin. It has thus far met with problems with infection and malfunction in initial trials, resulting in the need for frequent explanting [29]. Nevertheless, the more physiologic insulin delivery via the portal system (hepatic “first-pass”) and full automation based on accurate glucose sensing could be the equivalent of pancreas replacement technology of the future [30].

## 10. Towards Fully Automated Insulin Delivery

**Figure 11:**
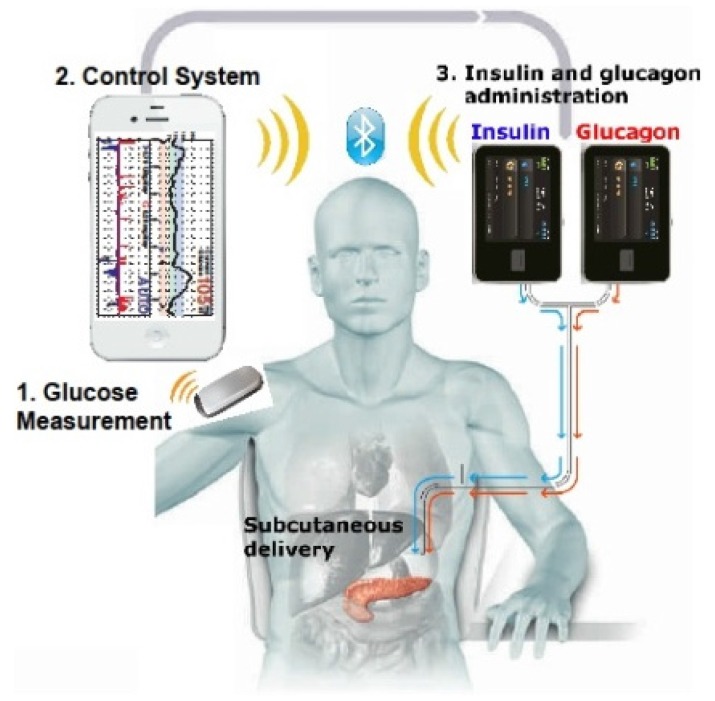
The bionic pancreas device system (available online: http://fortune.com/2014/06/16/apple-powered-bionic-pancreas-one-step-closer/, accessed on 3 December 2015).

The modest success of a “bionic pancreas” in type 1 diabetes was reported in a clinical study [31]. The researchers tested a bihormonal bionic pancreas in both adults and children using a removable tiny sensor located in a thin needle inserted under the skin that automatically monitored real time glucose levels in tissue fluid. It also provided insulin and the hormone glucagon, via two automatic pumps, while the patients carried a cell phone-sized wireless monitor for five days (Figure 11). For comparison, the patients were also monitored for five days wearing their own regular pumps that delivered insulin. The bionic system significantly reduced average blood glucose levels and required 37% less intervention for hypoglycemia. Although it validated the proof-of-concept for this type of therapeutic approach, the system needs further sophistication and testing over a sustained period of time. Technological advances pertaining to the use of closed-loop systems in conjunction with insulin pump therapy for type 1 diabetes are evolving [32].

## 11. Implementation of Pump Therapy

The following are some case-based approaches that illustrate the benefits and pitfalls of insulin pump therapy in clinical practice.

### 11.1. Case 1—Successful Pump Therapy Implementation

SQ is a 19 year old female with type 1 diabetes since the age of 12. She was referred by her pediatrician to transition care to adult endocrinology. She was hospitalized upon diagnosis for the treatment of diabetic ketoacidosis (DXA). In the first few years, SQ struggled to adjust to her new diagnosis. Despite frequent changes in her multiple daily insulin injection regimens, she continued to have erratic glycemic control, with HbA1c ranging from 8.6% to 10.8%. Her menstrual cycles started at age 13 and the patient had normal growth for her age; however, on her healthy child follow up visits, the patient frequently complained of chronic fatigue. Her school grades fell and her participation in extracurricular activities waned. While in high school, SQ’s parents enrolled her in a diabetes summer camp where she was taught how to count carbohydrates properly and learned the importance of good glycemic control along with her peers who had the same disease and challenges. SQ began checking her blood glucose prior to each meal and her blood sugars improved greatly with proper pre-meal bolusing. Concurrently, her energy level and grades improved, and she began participating in school activities again. Over the last year, her HbA1c has ranged from 7.4% to 7.8% and she has had no episodes of DKA. She is a counselor at the same diabetes camp that she attended. Now in college, she is interested in learning more about insulin pump therapy.

Questions:

1. Is SQ a good candidate for insulin pump therapy? Why or why not?
2. Does pump therapy offer better control than multiple daily insulin injections (MDII)?

Commentary: 

Factors predictive of successful implementation of pump treatment are outlined in Table 3. Careful patient selection is the single most important factor that predicts the success of insulin pump therapy. Diabetes during adolescence can make achieving glycemic control difficult. As a result, school performance and socialization can suffer. SQ was able to overcome these challenges and became an ideal candidate for insulin pump therapy. She is a proactive and knowledgeable patient who appears well-versed in the different aspects of daily diabetes self-care, including fingerstick monitoring and carbohydrate counting. She is motivated and desirous of initiating pump therapy. She undergoes pump training under the auspices of a professional team of experts, including the endocrinologist, trainer, and dietician. The physician determines her basal and bolus pump settings, carb ratio, sensitivity factor, and glucose targets. She checks her glucose readings 10–12 times a day during the first few weeks in order to fine-tune the pump settings and adjust to her new therapeutic management. In the months that follow, she finds that pump use is much more compatible with the hectic life of a college student. Her HbA1c drops to 6.9%. Thereafter, the patient continues to diligently check her blood glucoses 4–6 times a day and has only rare episodes of hypoglycemia. She is now contemplating obtaining a personal continuous glucose sensor for more focused glycemic management. Although optimal glucose control can be achieved with MDII, the pump offers flexibility, less insulin-glucose variability, and can be superior to MDII when expert guidance and follow-up is available [9].

**Table 3:** Factors related to Successful Initiation and Maintenance of Insulin Pump Therapy.

Availability of a team of health care professionals who have experience and proficiency in various aspects of pump use (endocrinologist/diabetologist, nurse educator, nutritionist, pump trainer)
Proper selection of candidates for pump therapy (motivated patient desirous of improved diabetes management who is regularly monitoring blood glucose and has realistic expectations of the benefits of pump use, is trained in pump technology, has learnt carbohydrate counting, and can troubleshoot issues)
Close communication and follow-up of the patient by the team of medical professionals in the time period immediately after pump initiation and in the long-term

### 11.2. Case 2—Alleviation of Hypoglycemia

LK is a 54 year old male with a history of type 1 diabetes for 31 years. He has been fairly well controlled on MDII, with an HbA1c of 7.2% during a recent office visit. However, the patient states that he was hospitalized two weeks ago after his wife noted what appeared to be seizure-like activity during sleep. EMS noted a glucose meter reading of 20 mg/dL. He reports glucose variability during the day with frequent hypoglycemia, but reports little or no symptoms. LK became very fearful of nocturnal hypoglycemia, and began checking his blood glucose between 2 and 4 a.m. He was shocked to find readings ranging from 30 to 50 mg/dL, often with no associated symptoms. He also reported high readings in the mornings. His MDII regimen was switched from NPH and regular insulin to glargine and aspart, with only minor reduction in hypoglycemic episodes.

Questions:

1. What are some of the challenge facing LK?
2. Can pump therapy minimize his risk of glycemic variability and nocturnal hypoglycemia?

Commentary: 

Older individuals with long-standing type 1 diabetes may develop defects in glucose counter-regulation, lack of adrenergic warning signals, and progressive hypoglycemia unawareness. Hypoglycemia-associated autonomic failure (HAAF) may supervene, and pose a particular challenge with regards to glycemic unpredictability and nocturnal hypoglycemia [33]. With a seemingly good HbA1c and lack of symptomatology, hypoglycemia can sometimes be missed; however, the patient’s elevated fasting morning readings should be a hint that he might be suffering from wide glucose fluctuations.

LK underwent a three-day professional CGM which revealed several daily episodes of hypoglycemia interspersed with acute elevations. Thus, his seemingly good HbA1c hid a large degree of glucose variability and hypoglycemic risk. LK was transitioned to an insulin pump and the settings were programmed and fine-tuned on the basis of his self-monitored readings and professional CGM. The latter showed that his hypoglycemia episodes had deceased considerably. He was able to reduce his HbA1c further to 6.8%, while concurrently minimizing hypoglycemia. Thereafter, he expressed an interest in, and was able to successfully procure, a personal CGM with real-time readings that integrated with the pump, with alarms and trend arrows [24]. The patient has expressed great relief in having this safety feature at his disposal. He has had only minor hypoglycemia and no further need for medical intervention. He qualifies for one of the new model insulin pumps with the “Threshold Suspend” feature [26].

### 11.3. Case 3—Pitfalls in Initiation and Use of the Insulin Pump

BF is a 33-year-old female who has been living with type 1 diabetes for over 16 years. She has had a difficult time adhering to lifestyle approaches, carbohydrate counting, and remembering to inject insulin. She has had several episodes of DKA requiring hospitalization over the course of her disease, with HbA1c ranging between 9.2% and 11.5%. She is a single mother who works two jobs to support her family and maintain her insurance coverage. When she found out that a coworker with diabetes was using an insulin pump, she decided to ask her primary care physician (PCP) about getting one for herself. She was convinced that the pump would make it vastly easier for her to manage her diabetes-related burden, rid her of insulin injections and glucose checks, and give her flexibility in what and when to eat. She was also under the impression that the pump would automatically take over the task of insulin delivery and function like a human pancreas—in her mind, the closest thing to a “cure” for her disease. Her PCP did little to dispel the patient’s notions. Instead, he readily complied with her request by writing a prescription for an insulin pump and supplies. A representative from a pump manufacturing company helped with submitting the paperwork and justifying the need for a pump for the patient. The representative met with the patient for a one-hour pump-initiation visit in a fast-food restaurant. Topics such as pump settings, carb counting, bolusing, and site changes were discussed in a summary fashion. The patient was instructed to contact the representative or the PCP’s office if she encountered “problems” during the first few weeks of pump use. However, without close supervision and trouble-shooting, the patient gradually reduced her fingerstick monitoring and could not carb count or bolus properly. Her dietary habits suffered and she felt she could eat whatever and whenever she wanted; all she had to do was to give herself an extra bolus of insulin via the pump. Her glycemic variability worsened and she started having very high glucose levels alternating with lows from insulin-stacking in a “roller-coaster” fashion. Her most recent HbA1c six months after switching to the pump was measured at 10.6%, and the patient has felt increasingly frustrated with her situation.

Questions:

1. What were the factors that led to lack of success with pump use in BF’s case?
2. What should be done to rectify the situation?

Commentary:

The elements predictive of the success of insulin pump therapy are reviewed in Table 3 above. BF had unrealistic expectations of pump therapy; she was neither educated nor well-trained for the transition; the PCP merely prescribed a pump reflexively without affording her the benefit of an expert team of professionals who would work closely with the patient before and after pump initiation. Contrary to prevalent misconception, the pump does not monitor blood glucose or automatically give insulin; in fact, it is incumbent that the patient check fingerstick readings several times a day (before and 2-h after meals, bedtime, 12:00 midnight, and 3:00 a.m., and any other times when necessary) and communicate these daily for several weeks with the physician’s office; the latter should be a diabetologist or endocrinologist with experience in managing patients treated with pumps. The patient will need refresher classes in carb counting and the logistics of pump use. The pump is programmable and does offer flexibility; however, it does not have a “mind” of its own and is only as good as the user wearing it. Many patients fall into a pattern of dietary indiscretion and over-eating by “learning” to vary the amounts and frequency of insulin boluses to cover large amounts of carbohydrates and snacks, resulting in weight gain, hyperglycemic peaks, and hypoglycemic episodes. Like any other therapeutic tool, the pump can be misused and abused [34]. The patient started with pump therapy on the wrong footing and felt completely lost and without any guidance. The result was a “train-wreck” disaster with frustratingly worse outcomes than prior to initiating the pump.

The patient was subsequently referred to an endocrinologist’s office and was offered two options. She could go back to MDII in order to break the deleterious cycle of continuing with her current level of understanding and usage of the pump without accruing any of its potential benefits. The other alternative would be to practically start from scratch and undergo extensive retraining in the concept and proper use of insulin pump therapy, including a realistic assessment of its pros and cons and the effort required to successfully maintain pump use over the long-term. The patient chose to “take a break” and switch back to MDII with the possibility of re-initiating the pump in a proper manner at a future time.

### 11.4. Case 4—Advantages of the Insulin Pump in Pregnancy

MT is a 34 year old female with type 1 diabetes who is planning to undergo *in vitro* fertilization (IVF) to become pregnant with her third child; she presents to the physician’s office for recommendations regarding improved glycemic control. She was diagnosed with type 1 diabetes in her early 20s. She admits that she did not take her diagnosis seriously with her first pregnancy, and had an average HbA1c in the 11% range. Unfortunately, her first child was born with birth defects. During her second pregnancy, MT made a concerted effort to abide by the multiple daily insulin injection regimen outlined by her PCP, resulting in an average HbA1c 7.5%–8.5%. She delivered a health child at 35 weeks’ gestation. She is now planning her third pregnancy and is nervous about the well-being of the fetus. She wants advice on improving her glycemic control.

Questions:

1. Is insulin pump therapy a good option during pregnancy?
2. What are the glycemic targets for women with preexisting diabetes who become pregnant?

Commentary: 

Following diabetic education (including carbohydrate counting classes), MT is initiated on insulin pump therapy. She is able to achieve and maintain an HbA1c 6.1%–6.3% prior to the planned conception by IVF, and delivers a healthy baby boy at 37 weeks’ gestation. Pregnant women with type 1 diabetes may be able to achieve better control with insulin pump therapy compared to other treatments, including insulin injections [35]. Optimal glucose control should be instituted with proper pre-conception planning, including the initiation of pump therapy in appropriately selected patients with both type 1 and type 2 diabetes. The Endocrine Society Guidelines [36] recommend the following stringent glycemic goals for preexisting diabetes who become pregnant: before meals (preprandial): <95 mg/dL, morning/fasting: <90 mg/dL, and after meals (postprandial): <120 mg/dL. The HbA1c goal during pregnancy is <6.5%.

### 11.5. Case 5—Perioperative Management in Patients Using an Insulin Pump

MH is a 66-year-old patient with type 1 diabetes who is undergoing surgical evaluation for right knee arthroplasty. He is a long time marathon runner and has been well controlled on continuous subcutaneous insulin infusion (CSII) therapy for the last 12 years. His average HbA1c ranged from 6.7% to 7.2% over the last year. Two years ago, the patient underwent left knee arthroplasty; his insulin pump was discontinued the morning of surgery on instructions from the surgical nurse. The operation lasted a little over five hours and the patient was sent to the postoperative area without restarting the pump. Postoperatively, the patient was noted to have fingerstick blood glucose of 277 mg/dL and an elevated anion gap. His hospital stay had to be extended due to treatment for hyperglycemia and mild DKA. Eventually, the CSII was restored with assistance from the inpatient diabetes educator. With this experience, MH wishes to be proactive regarding his upcoming surgery and would like your recommendations regarding insulin pump management.

Question:

How should patients on insulin pump therapy be managed during elective or emergent surgery?

Commentary: 

Patients on continuous subcutaneous insulin infusion, or insulin pumps, are increasingly seen in hospitals. Given the heterogeneity of surgical procedures and patients, there is no standardized approach to managing insulin pumps in this setting. An individualized approach should be taken [37]. When there is time for planning, as in elective procedures, tailored recommendations can be made. As much as possible, the input and advice of the specialist who manages the patient’s pump should be sought and followed. A central guiding principle to be kept in mind is: *Do the circumstances of the surgery allow the patient to be awake, alert, and be able to reliably operate the pump (other than procedures lasting only a few hours)?* The assumption is that the patient is in the best position to operate his/her own pump. It is desirable to have the endocrinologist or an inpatient diabetes specialist with thorough expertise in pump management available for advice. In general, the following factors need to be considered:

- The duration of the surgical procedure: in longer surgeries, the pump should be disconnected and the patient treated with injections or an insulin drip. Procedures of short duration (2–3 h) can be managed by keeping the pump in place and possibly employing a reduced temporary basal rate for several hours.
- Whether the procedure is elective or emergent: in the latter situation it is almost always advisable for the clinician to take over glycemic management by discontinuing the pump and substituting correctional or intravenous insulin.
- The type of anesthesia (general, local, or sedation): obviously, general anesthesia or sedation will not permit the patient to have input in using the pump.
- Will the patient need to be moved and is the pump insertion site at risk of becoming disconnected? If so, it may be more prudent to disconnect the insulin pump and use intravenous insulin infusion to control blood sugar levels. Also, pump manufacturers recommend that pumps be placed out of range of radiation.

It was decided that MH would stay on his insulin pump during his knee surgery at a slightly reduced basal rate, as determined on recommendations from his endocrinologist, who was also available for phone or in-person consultations if necessary. The operation went extremely well, and MH’s hourly fingerstick readings ranged between 117 and 143 mg/dL. The patient able to resume oral feedings and manage his pump boluses within an hour after the surgery was over.

### 11.6. Case 6—Benefits of the Insulin Pump in Patients with Type 2 Diabetes and Insulin Resistance

RW is a 47-year-old Hispanic male who has had type 2 diabetes for 16 years. He is morbidly obese with a body-mass index (BMI) of 43. Both of his parents and two of his three siblings suffer from diabetes. He is taking metformin and multiple-dose insulin injections. He has tried incretin-based therapies in the past for their glucose-lowering and weight-stabilizing benefits but could not tolerate the gastrointestinal side-effects. The patient’s daily insulin dose has been progressively increased to a regimen of glargine 115 units twice a day and aspart 45–60 units prior to each meal. He prefers to use disposable insulin pens for convenience; however, individual pens only hold 300 units and do not last more than two days each. Efforts at lifestyle changes aimed at weight loss have been marginally successful. His A1c level varies between 7.9% and 8.6%. He is motivated to improve his health and checks his fingerstick glucose readings several times a day.

Question:

Considering his high insulin requirements and burden of multiple daily insulin injections, what management options could be presented to him?

Commentary:

Although most of the earlier data on CSII was gained from use of insulin pumps in children and young adults with type 1 diabetes, this method of treatment has gained popularity in patients with type 2 diabetes as well. The latter seem to benefit from the recognized advantages of pumps with regard to a more physiologic insulin replacement regimen and better lifestyle acceptability [38]. Type 2 patients who are on a titrated MDII regimen and have suboptimal glycemic control can achieve lower A1c level with pump therapy [39]. Patients who have extremely elevated insulin resistance, as signified by high insulin requirements (more than 200 units per day), central obesity, and concomitant hypertension and dyslipidemia, *i.e.*, a metabolic syndrome phenotype, frequently face challenges in improving glucose control in spite of titration of insulin dosage. The patient described in this case scenario refused to consider bariatric surgery. One option is to use a more concentrated insulin formulation, such as regular U500 insulin three times a day at mealtimes; however, although the volume of injected insulin is significantly reduced, a true basal-bolus pattern is difficult to achieve with this approach. After an informed discussion, RW was initiated on pump therapy using U500 insulin [40]. He required only one-fifth as much volume as U100 insulin, thus permitting larger daily amounts to be delivered to overcome insulin resistance. It also allowed him to replenish insulin once every 3 days rather than on a daily basis. With the combination of an insulin pump and U500 insulin, he was able to achieve an A1c of less than 7% while obtaining freedom from seven to eight insulin injections every day. He also evinced greater treatment satisfaction and better quality of life.

## 12. Final Thoughts

The last quarter century has witnessed remarkable advances in replicating the natural pancreas function with insulin pump devices based on increasingly accurate sensing, precise delivery, reduction in glycemic excursions, and inherent safeguards against hypoglycemia. While systems with more complexity are likely to emerge, they promise to mimic the amazingly multifaceted coordination of glucose control found in the natural state through the intricate use of automated precision technology. The proper education of motivated and knowledgeable patients, supported by an experienced team of health care professionals, gives the best outcomes in reducing the burden of hyperglycemia as well as hypoglycemia. Careful selection of patients who will predictably do well with continuous insulin delivery is perhaps the most important factor in this regard. For ongoing management utilizing an insulin pump, the availability of expertise to appropriately guide patients and promptly troubleshoot issues is essential. Successful translation of technological advances to clinical care poses challenges and opportunities for providers and patients alike.
